# Recent COVID-19 Infection Increases Complication Risk After Body-Contouring Surgery

**DOI:** 10.1093/asjof/ojaf141

**Published:** 2025-10-31

**Authors:** Matthew Q Dao, Diana Shaari, Abigail R Tirrell, Brooke Barrow, Sheuli Chowdhury, Bernice Z Yu, Paul Won, BaiJing Qin, Peter J Taub, Peter W Henderson

## Abstract

**Background:**

Although COVID-19 infection rates have declined from pandemic peaks, recent infection may pose a potential concern in aesthetic surgery. Of note, the surgical risks associated with recent infection are not well defined. Previous studies, constrained by small cohorts and early-pandemic data, have not conclusively established whether recent COVID-19 infection continues to influence surgical outcomes.

**Objectives:**

The authors sought to determine whether COVID-19 infection within 30 days before body-contouring procedures is associated with increased postoperative complications.

**Methods:**

Adult patients who underwent body-contouring surgery between August 2020 and March 2025 were identified from the US Collaborative Network on TriNetX (TRINETX, LLC, Cambridge, MA). Patients were categorized based on documented COVID-19 infection within 30 days preoperatively. Propensity score matching (1:1) was performed to balance demographics and comorbidities. Thirty-day postoperative complications were compared using risk ratios (RRs), with statistical significance defined as *P* < .05.

**Results:**

A total of 3941 patients were matched in each of the COVID-19 and non-COVID-19 groups. Patients in the matched COVID-19 group had a significantly increased risk of surgical-site infection (RR 1.56, *P* = .010), wound disruption (RR 1.69, *P* = .003), postoperative pain (RR 1.66, *P* = .002), anticoagulant use (RR 1.77, *P* < .0001), and emergency department visits (RR 1.50, *P* = .010).

**Conclusions:**

Despite reduced overall prevalence, recent COVID-19 infection remains associated with increased risk of postoperative complications following body-contouring surgery. Delaying elective aesthetic procedures by at least 30 days following recent infection appears prudent to enhance patient safety and minimize complications.

**Level of Evidence: 3 (Therapeutic):**

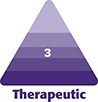

The COVID-19 pandemic significantly disrupted elective surgical procedures worldwide, including aesthetic surgery. After initial lockdown restrictions were lifted, procedural demand surged across many regions.^1-4^ As operations resumed, institutions implemented varied preoperative screening protocols for COVID-19, resulting in inconsistent practices nationwide.^5^ Although infection rates have declined, COVID-19 has not been eradicated and continues to pose a risk, reinforcing the importance of vigilance in surgical planning.

Body-contouring surgery is commonly performed to address excess skin and contour deformities following massive weight loss, often after bariatric surgery. These deformities can impair body image, functional mobility, and overall quality of life.^6^ Procedures, including abdominoplasty, panniculectomy, brachioplasty, and thighplasty, aim to improve aesthetic appearance, physical function, and psychosocial well-being.^7^ According to the American Society of Plastic Surgeons, the demand for body-contouring procedures has steadily increased; post–weight loss operations exceeded 46,000 in 2020.^8^ Annual totals for aesthetic procedures have continued to rise, with ∼348,000 liposuction cases by 2023 and 171,000 abdominoplasties by 2024.^9,10^ These rising numbers reaffirm the relevance of body contouring in the postpandemic era, as aesthetic procedures are increasingly prioritized by patients.

Early studies examining the effects of COVID-19 on postoperative outcomes suggest an increased risk of complications. Duggan et al reported elevated rates of wound disruption, reoperation, and hospital readmission among patients undergoing breast or body-contouring surgery within 3 months of COVID-19 infection.^11^ Similarly, Wang et al observed delayed wound healing in patients undergoing abdominoplasty with any history of previous COVID-19 infection.^12^ However, these studies were limited by small sample sizes, early-pandemic populations, and broad definitions of infection timing, restricting their applicability to current clinical practice.

Given the elective nature of aesthetic surgery and ongoing uncertainty regarding the perioperative implications of recent COVID-19 infection, more robust evidence is needed to guide clinical decision making. Therefore, the objective of the authors of this study is to determine whether documented COVID-19 infection within 30 days before body-contouring surgery is associated with increased postoperative complications. By addressing this clinical uncertainty, the present analysis aims to inform perioperative risk assessment and support the broader integration of infectious disease considerations into aesthetic surgical practice.

## METHODS

### Data Source

For this retrospective cohort study, the authors utilized data from the TriNetX research platform (TRINETX, LLC, Cambridge, MA), a global, federated network designed for real-time access to aggregated and de-identified electronic health records. On April 1, 2025, data were queried from the United States Collaborative Network within TriNetX, which comprises clinical data contributed by 67 healthcare organizations, including academic medical centers, community hospitals, and outpatient clinics. This extensive network encompasses over 120 million unique patient records, offering a robust sample for diverse population research. Given that all patient information is anonymized and compliant with Health Insurance Portability and Accountability Act standards before researcher access, this analysis met criteria for exemption from IRB oversight.

### Cohort Selection

Adult patients aged 18 years and older who underwent body-contouring surgery between August 2020 and March 2025 were identified using Current Procedural Terminology (CPT) codes (Supplemental Table). This 5-year time interval was intentionally selected to capture outcomes across multiple phases of the COVID-19 pandemic and into the postpandemic era, allowing for a more comprehensive analysis of COVID-19's lasting impact on surgical risks. Body-contouring procedures included excision of excess skin and subcutaneous tissue from the submental region, arms, forearms, hands, abdominal wall (both abdominoplasty and panniculectomy), hips, buttocks, thighs, and legs, as well as mastopexy and mastectomy for gynecomastia. All included CPT codes were collectively pooled to form the body-contouring cohorts.

COVID-19 diagnoses were identified using International Classification of Diseases, 10th Revision (ICD-10) and Logical Observation Identifiers Names (Logical Observation Identifiers Names codes; Supplemental Table). Eligible patients were categorized into 2 groups based on COVID-19 infection status: those with a documented COVID-19 diagnosis within 30 days before surgery (unmatched COVID-19 group) and those without (unmatched non-COVID-19 group). This 30-day preoperative window was selected to specifically assess the effects of recent infection on postoperative outcomes. Patients included in this study were determined by a clinically diagnosed ICD-10 code or confirmed laboratory testing for RNA presence of the virus.

### Propensity Score Matching

**Figure 1.**
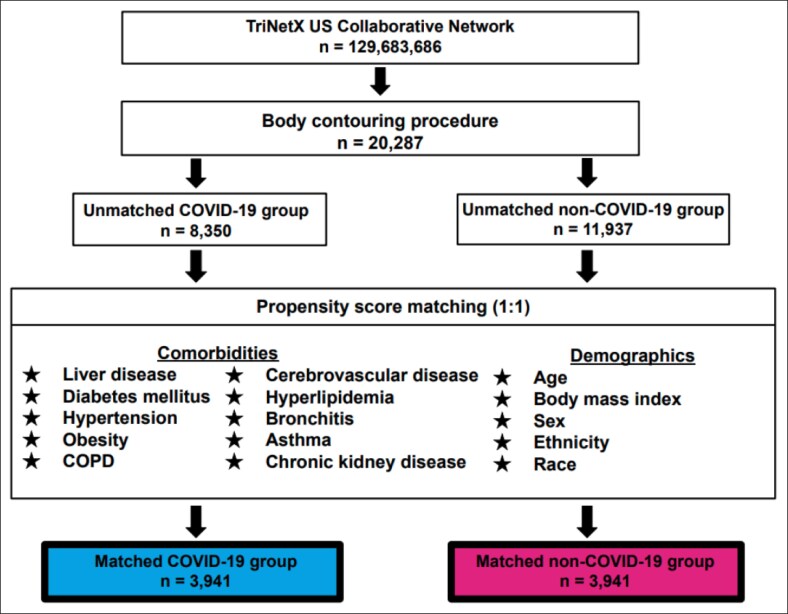
Diagram outlining study design. Data were extracted from the US Collaborative Network within TriNetX, encompassing 69 healthcare organizations. An initial cohort of 20,287 body-contouring patients was identified, with 3941 patients retained in both the COVID-19 and matched control groups following 1:1 propensity score matching.

A 1:1 propensity score matching approach was employed using logistic regression and a nearest-neighbor algorithm. To reduce the influence of confounding factors, patients with recent COVID-19 infection were matched to those without documented infection based on key demographic variables (age, sex, BMI, race, and ethnicity) and clinically relevant comorbidities, including liver disease, diabetes mellitus, hypertension, obesity, chronic obstructive pulmonary disease (COPD), cerebrovascular disease, hyperlipidemia, bronchitis, asthma, and chronic kidney disease. Covariate balance was considered adequate when postmatching comparisons yielded *P*-values >.05, indicating no statistically significant differences between groups remained. The final matched cohorts were referred to as the matched COVID-19 group and the matched control group. A visual overview of the study design is presented in Figure 1.

### Outcomes

Postoperative outcomes were classified as either surgical or nonsurgical complications to distinguish between local wound-related events and systemic or care-related issues. Surgical complications were defined as those involving the operative site or directly related to tissue healing, including surgical-site infection (SSI), wound disruption, hematoma, and seroma. Nonsurgical complications included acute postoperative pain, anticoagulant use (eg, rivaroxaban, warfarin, apixaban, heparin, enoxaparin, among others), emergency department (ED) visits, and postoperative anemia (Supplemental Table). All outcomes were assessed within a 30-day postoperative window to capture acute morbidity associated with recent preoperative COVID-19 infection.

### Statistical Analysis

All statistical analyses were conducted using the TriNetX analytics platform, which integrates multiple programming environments, including JAVA, R, and Python, to facilitate comprehensive data processing and analysis. Continuous variables were compared using Student's *t*-test and presented as means with standard deviations. Categorical variables were assessed using either the χ^2^ test or Fisher's exact test, as appropriate.

Multiple univariate regression models were utilized to assess differences in each postoperative outcome. Effect estimates were expressed as risk ratios (RRs) with corresponding 95% CIs. Statistical significance was defined as a 2-tailed *P*-value <.05.

### Compliance With Reporting Standards

This investigation was conducted in alignment with the Strengthening the Reporting of Observational Studies in Epidemiology (STROBE) guidelines, which provide a structured framework for reporting cohort studies and other observational research.^13^ These recommendations aim to improve the quality, transparency, and reproducibility of observational studies by outlining key elements that should be included in study design, conduct, analysis, and interpretation. By following the STROBE principles, the authors are committed to providing a clear and reproducible account of the study's design, data analysis, and interpretation.

## RESULTS

### Baseline Characteristics Before and After Matching

An initial cohort of 20,287 patients who underwent body-contouring surgery was identified. Before propensity score matching, 8350 patients had a documented COVID-19 infection within 30 days preoperatively, whereas 11,937 served as unmatched controls with no history of recent infection. Patients in the unmatched COVID-19 group ranged in age from 18 to 90 years, with a mean age of 51 ± 14 years, compared with a mean age of 49 ± 14 years in the unmatched non-COVID-19 group. Mean BMI was also higher in the COVID-19 group (30.1 ± 7.09 kg/m^2^) relative to controls (29.2 ± 6.59 kg/m^2^). Across both unmatched groups, the predominant racial demographic was White, comprising 66.4% of the COVID-19 group and 60.7% of the control group (*P* < .001). Before matching, patients in the unmatched COVID-19 group exhibited significantly higher rates of several comorbidities, including liver disease (11.2% vs 6.7%), diabetes mellitus (17.0% vs 12.5%), hypertension (35.0% vs 27.2%), obesity (44.0% vs 32.0%), COPD (3.1% vs 1.9%), cerebrovascular disease (3.7% vs 2.3%), hyperlipidemia (22.7% vs 16.6%), bronchitis (5.0% vs 2.1%), asthma (18.0% vs 12.4%), and chronic kidney disease (4.6% vs 2.7%; all *P* < .001). After 1:1 propensity score matching based on demographic and clinical covariates, 3941 patients remained in each well-balanced group for outcome analysis. Postmatching, there were no statistically significant differences in baseline demographics or comorbidities between groups (all *P* > .05), indicating adequate control for covariates (Tables 1, 2).

**Table 1.** 1:1 Before-and-after Propensity Score Matching for Patient Demographics

Characteristics	Before matching		*P*-value	After matching		*P*-value
	Unmatched COVID-19 group *n* = 8350	Unmatched control group *n* = 11,937		Matched COVID-19 group *n* = 3941	Matched control group *n* = 3941	
Mean age ± SD	50.9 ± 14.0	49.4 ± 14.4	<.001	50.1 ± 14.3	50.1 ± 14.4	.979
Female	6983 (83.6%)	8948 (75.0%)	<.001	3255 (82.6%)	3264 (82.8%)	.117
Male	1348 (16.1%)	1900 (15.9%)	<.001	667 (16.9%)	656 (16.6%)	.740
Unknown sex	19 (0.5%)	1089 (0.5%)	<.001	19 (0.5%)	21 (0.5%)	.751
White	5543 (66.4%)	7250 (60.7%)	<.001	2612 (66.3%)	2631 (66.8%)	.650
American Indian	28 (0.3%)	48 (0.4%)	<.001	16 (0.4%)	18 (0.5%)	.731
Native Hawaiian or other Pacific Islanders	54 (0.6%)	56 (0.5%)	.003	22 (0.6%)	25 (0.6%)	.661
Black or African American	1376 (16.5%)	1531 (12.8%)	<.001	591 (15.0%)	596 (15.1%)	.875
Asian	230 (2.8%)	253 (2.1%)	<.001	104 (2.6%)	78 (2.0%)	.051
Other race	410 (4.9%)	621 (5.2%)	<.001	210 (5.3%)	195 (4.9%)	.444
Unknown race	709 (8.5%)	2178 (18.2%)	<.001	386 (9.8%)	398 (10.1%)	.652
Hispanic or Latino	1187 (14.2%)	1208 (10.1%)	<.001	481 (12.2%)	473 (12.0%)	.782
Not Hispanic or Latino	6512 (78.0%)	7340 (61.5%)	.010	2914 (73.9%)	2860 (72.6%)	.169

SD, standard deviation.

**Table 2.** Before and After 1:1 Propensity Score Matching for Comorbidities

Comorbidities	Before matching		*P*-value	After matching		*P*-value
	Unmatched COVID-19 group *n* = 8350	Unmatched control group *n* = 11,937		Matched COVID-19 group *n* = 3941	Matched control group *n* = 3941	
Liver disease	933 (11.2%)	801 (6.7%)	<.001	350 (8.9%)	310 (7.9%)	.104
Diabetes mellitus	1421 (17.0%)	1497 (12.5%)	<.001	545 (13.8%)	535 (13.6%)	.743
Hypertension	2924 (35.0%)	3244 (27.2%)	<.001	1146 (29.1%)	1139 (28.9%)	.315
Obesity	3676 (44.0%)	3825 (32.0%)	<.001	1462 (37.1%)	1419 (36.0%)	.315
COPD	262 (3.1%)	230 (1.9%)	<.001	100 (2.5%)	84 (2.1%)	.233
Cerebrovascular diseases	308 (3.7%)	277 (2.3%)	<.001	114 (2.9%)	107 (2.7%)	.633
Hyperlipidemia	1892 (22.7%)	1983 (16.6%)	<.001	741 (18.8%)	720 (18.3%)	.543
Bronchitis	415 (5.0%)	249 (2.1%)	<.001	139 (3.5%)	117 (3.0%)	.162
Asthma	1507 (18.0%)	1484 (12.4%)	<.001	557 (14.1%)	570 (14.5%)	.676
Chronic kidney disease	387 (4.6%)	324 (2.7%)	<.001	145 (3.7%)	133 (3.4%)	.464

COPD, chronic obstructive pulmonary disease.

### Surgical-Site Complications

**Figure 2.**
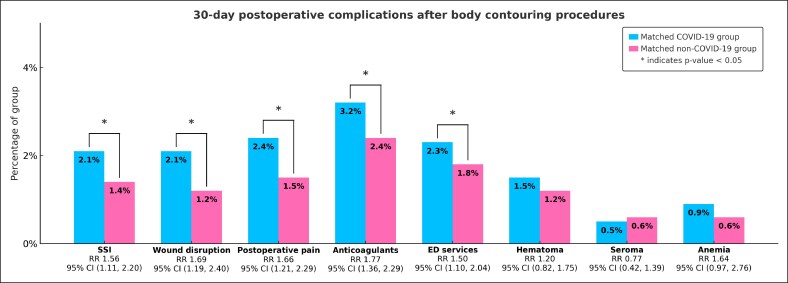
Thirty-day postoperative complications in patients who underwent body-contouring procedures with and without recent preoperative COVID-19 infection. RR, risk ratio; SSI, surgical-site infection; ED, emergency department. Percentage of group (%) represents proportion of patients in each matched group experiencing outcome.

Following propensity score matching, patients in the matched COVID-19 group demonstrated significantly elevated risks for postoperative complications within 30 days of body-contouring surgery. Rates of SSI (RR 1.56, 95% CI, 1.11-2.20, *P* = .010) and wound disruption (RR 1.69, 95% CI, 1.19-2.40, *P* = .003) were significantly higher compared with matched non-COVID-19 group. Acute postoperative pain was also more common in the matched COVID-19 group (RR 1.66, 95% CI, 1.21-2.29, *P* = .002). No statistically significant differences were observed for hematoma and seroma (Figure 2).

### Nonsurgical and Systemic Events

Beyond local wound outcomes, the matched COVID-19 groups were more likely to require postoperative anticoagulant therapy (RR 1.77, 95% CI, 1.36-2.29, *P* < .0001). ED visits were also significantly higher among the matched COVID-19 group (RR 1.50, 95% CI, 1.10-2.04, *P* = .010). Postoperative anemia did not differ significantly between the matched groups (Figure 2).

## DISCUSSION

This nationwide study is the largest matched cohort analysis to date evaluating postoperative outcomes among patients with recent COVID-19 infection undergoing body-contouring surgery. Using a large, real-world dataset and a rigorously defined preoperative infection window, the authors of this study identified heightened risks for SSI, wound disruption, postoperative pain, anticoagulant use, and ED utilization in patients diagnosed with COVID-19 within 30 days before surgery. These findings therefore emphasize the importance of considering recent COVID-19 infection as a clinically relevant preoperative risk factor in body-contouring procedures.

SSI occurred with greater frequency in patients who underwent body-contouring procedures within 30 days of a COVID-19 diagnosis, highlighting the lingering immunologic and vascular consequences of the viral infection.^14,15^ This finding contrasts with the earlier study by Duggan et al, which reported no significant difference in postoperative infection rates between COVID-19 and control cohorts.^11^ However, that previous analysis had included patients with a history of COVID-19 infection up to 3 months before body-contouring surgery, which may have obscured the complication risks. Emerging evidence have indicated that COVID-19 can drive prolonged immune dysregulation even after resolution of acute symptoms.^16^ As such, impaired neutrophil chemotaxis and persistently elevated cytokines, such as IL-6, TNF-α, and IL-1β, can weaken antimicrobial defenses at surgical sites.^17^ Thus, this compromised immune surveillance of the skin may, in turn, increase susceptibility to local infection.^18-20^ Additionally, the prothrombotic and hypoxic microenvironment induced by COVID-19 may impair capillary perfusion and oxygen delivery to healing tissues, which could further enhance bacterial growth.^21^

Wound disruption occurred at a significantly higher rate in patients who underwent body-contouring procedures within 30 days of a COVID-19 diagnosis, indicating the virus's detrimental effects on tissue repair. COVID-19 has been shown to prolong the inflammatory phase of wound healing, leading to dysregulated expression of matrix metalloproteinases and attenuated fibroblast function.^22^ These disruptions may impair collagen deposition and extracellular matrix remodeling, which are essential for restoring tensile strength.^23^ Concurrently, attenuated transforming growth factor-beta signaling and fibroblast senescence may delay tissue formation and hinder re-epithelialization.^24,25^ Consequently, delayed wound healing from endothelial dysfunction and poor oxygen delivery creates a suboptimal environment for repair.^26,27^ This study's finding is consistent with previous studies by Duggan et al and Wang et al, both of which reported delayed wound healing in COVID-19-positive patients undergoing body-contouring procedures.^11,12^ Such a complication is especially important in surgeries involving extensive tissue manipulation, where wide excisions and high-tension closures may exacerbate the risk of wound disruption in patients with COVID-19. Collectively, these findings warrant heightened postoperative vigilance for wound complications in patients with recent COVID-19 infection.

The increased incidence of postoperative pain observed among patients with recent COVID-19 infection potentially reflects neurobiological disruption associated with the virus. COVID-19 has been shown to promote neuroinflammation through activation of glial cells and persistent upregulation of proinflammatory cytokines within the peripheral and central nervous systems.^28,29^ These mediators can sensitize nociceptors and lower pain thresholds, contributing to hyperalgesia and neuropathic pain states.^30^ Moreover, dysregulation of angiotensin-converting enzyme 2 signaling in dorsal root ganglia may further amplify nociceptive transmission and hinder recovery from surgical pain.^31^ Several studies have documented “long COVID” phenotypes involving persistent myalgias, arthralgias, and sensory hypersensitivity, suggesting sustained disruption of pain modulation pathways.^32,33^ In the postoperative period, these neuroinflammatory changes may manifest as heightened analgesic requirements, prolonged pain trajectories, or atypical pain presentations. Nevertheless, further research is encouraged to clarify the duration and to guide targeted pain management in recently infected patients undergoing surgery.

The elevated rate of postoperative anticoagulant use in patients with recent COVID-19 infection likely reflects growing awareness of the virus's prothrombotic effects.^34^ Notably, the COVID-19 virus has been reported to increase circulating D-dimer and fibrinogen levels, which may promote a hypercoagulable state.^35,36^ Accordingly, hypercoagulability may further be exacerbated in body-contouring patients, where obesity, extensive tissue dissection, and postoperative immobility could amplify thrombotic risk.^37,38^ Because this study's dataset does not distinguish between prophylactic and therapeutic anticoagulation, the disproportionately high rate of anticoagulant use in the COVID-19 group may also reflect physician-initiated strategies to manage suspected or confirmed venous thromboembolism (VTE).^39^ As such, anticoagulant use could be interpreted as a surrogate marker of thrombotic risk rather than a direct measure of VTE. Nonetheless, the higher postoperative utilization of anticoagulants in the matched COVID-19 cohort reinforces an ongoing concern for thrombotic complications in this population. These findings align with previous reports of delayed thrombosis and support thromboprophylaxis in recently infected body-contouring patients.^40^

ED utilization within 30 days of surgery was significantly elevated in patients with recent COVID-19 infection. This increase is likely attributable to greater incidence of postoperative complications, including wound disruption, SSI, and postoperative pain, which naturally necessitate more frequent unplanned evaluations. In addition, persistent physiologic derangements associated with COVID-19, including endothelial injury, cardiopulmonary compromise, and autonomic instability, may present as dyspnea, chest discomfort, or palpitations during the early postoperative period.^36,41,42^ As mentioned, long COVID has been increasingly recognized as a syndrome of sustained inflammation and multiorgan dysfunction, affecting the cardiovascular, pulmonary, renal, and neurologic systems.^43^ These lingering effects can amplify postoperative morbidities and prompt additional emergency care. As such, the elevated ED utilization rate highlights the systemic burden imposed by recent infection and underscores the need for structured follow-up and anticipatory management protocols in this patient population.

### Limitations

This study possesses several noteworthy strengths. The large patient sample size provides robust statistical power to detect differences in both common and uncommon postoperative complications, whereas the multicenter design enhances the external validity of the findings across diverse US populations. Additionally, the breadth and depth of clinical data available through TriNetX enable meaningful subgroup analyses across a wide range of demographics, comorbidities, and healthcare settings. Although retrospective in nature, the use of rigorous 1:1 propensity score matching minimized the impact of baseline confounders and improved comparability.

Nevertheless, several important limitations should be considered. As with all retrospective analyses, confounding elements from unmeasured clinical variables could not be controlled for from the electronic health records. First, the TriNetX platform does not include details on COVID-19 symptom severity, timing of resolution, viral strain, booster timing, method of viral testing, or vaccination status, which may be factors that influence perioperative risk. As such, the COVID-19 diagnoses do not provide specific details on whether the patients were symptomatic, tested positive because of PCR results regardless of symptoms, or coded based on clinical suspicion alone. Furthermore, the absence of vaccination data limits the generalizability of the study given the likely widespread availability of vaccines during the study period and may lead to underestimation of protective capabilities in immunized individuals. Therefore, the authors encourage a follow-up study be conducted on assessing the impact of vaccination status in COVID-19 patients and outcomes following body-contouring procedures. Currently, these limitations from broad or vague classifications may restrict the ability to denote precise risk differences. Greater symptom burden or a shorter illness-to-surgery interval could increase risk of complications, whereas vaccination may reduce it. Second, outcomes were identified using ICD-10 codes, which limits granularity. For instance, wound disruptions could not be categorized as superficial vs deep, potentially obscuring clinically relevant distinctions. Third, although inclusion of a broad range of body-contouring procedures enhances generalizability, the authors were unable to perform stratified sub-analyses by procedure type (eg, abdominoplasty vs thighplasty). The authors admit this procedural heterogeneity poses as a limitation, as each surgery has varying risk profiles. This study pooled several elective body-contouring operations (eg, abdominoplasty or panniculectomy, mastopexy, brachioplasty, thighplasty, and gynecomastia surgery) into each cohort and could not stratify or match by CPT-defined procedure. Additionally, differences in case mix may have contributed to outcome variation; for example, a greater proportion of abdominoplasty cases in the COVID cohort vs more mastopexies in the non-COVID cohort could influence anticoagulant prescribing through Caprini-based risk stratification. Consequently, the present results showcase the overall association between recent COVID-19 infection and 30-day outcomes, which should not be interpreted as procedure specific. Therefore, the authors of further studies should evaluate more homogeneous cohorts undergoing a specific body-contouring procedure with standardized capture of extent and technique. Fourth, the dataset also lacks surgeon-specific and institution-level variables, such as operative technique, experience, and case volume, which could affect outcomes for patients. Future prospective studies at the institutional level should stratify patients by vaccination history, symptom burden, and infection severity, while also accounting for differences in surgeon experience and practice protocols. Lastly, although this study focused on a 30-day preoperative infection window, this time interval is broad. The authors of future studies should therefore evaluate surgical outcomes with more defined intervals from diagnosed infection status (eg, <30, 31-60, >90 days) and provide more precise guidance on optimal surgical timing.

### Clinical Implications

Despite these limitations, these findings carry important clinical implications for perioperative planning in aesthetic surgery. Patients with recent COVID-19 infection face significantly increased risks of postoperative infection, wound disruption, and acute pain, which conveys the need for cautious surgical timing. Although the prevalence of COVID-19 has markedly declined, its continued presence at lower community transmission rates reinforces the need to consider recent infection as a pertinent perioperative risk factor. However, the relevance of a recent COVID-19 diagnosis should not be confined to the pandemic era; this study's results support treating recency of infection as that may influence perioperative risk in aesthetic procedures. Currently, preoperative risk assessment does not require universal screening. Based on these findings, a brief preoperative inquiry about recent COVID-19 diagnosis or positive test within the previous 30 days could be considered; if present, deferring body-contouring surgery for at least 30 days may be a reasonable decision. Selective testing for symptomatic patients may also represent a cautious strategy under clinician discretion. This approach remains important as testing practices evolve because recent viral illness has been associated with impaired healing, increased thrombotic risk, and higher postoperative healthcare utilization. Furthermore, this study provides a framework for considering infectious disease history as a potentially relevant factor in perioperative risk stratification for patients.

Previous studies in general and orthopedic surgery have reported improved outcomes when elective procedures are delayed after COVID-19 infection; however, optimal timing remains uncertain, with recommendations ranging from 4 to 7 weeks depending on patient-specific factors, such as symptom severity, comorbidities, and type of procedure.^44-46^ Results from the present study support the possibility that a minimum 30-day delay following COVID-19 infection may help reduce complications, although further studies are needed to confirm the ideal interval. This timeframe, backed by significantly elevated complication rates observed in this analysis, offers surgeons a practical and evidence-based threshold for reducing postoperative risks.

For patients who cannot defer surgery, the authors recommend individualized risk mitigation that includes enhanced wound surveillance, early postoperative follow-up, and consideration of VTE prophylaxis in higher-risk cases. Considering recent infectious disease history in preoperative screening, much like smoking, could strengthen risk assessment. Moreover, delaying elective body-contouring procedures in patients recently infected with COVID-19 may also reduce healthcare utilization by lowering unplanned ED visits and complication rates. As aesthetic surgery continues to evolve, these measures will be essential in refining risk stratification and improving the quality of practice. Ultimately, maintaining perioperative vigilance regarding recent infectious diseases, such as COVID-19, remains important for patient safety and optimizing surgical outcomes.

## CONCLUSIONS

The authors of the present study report that COVID-19 infection within 30 days before body-contouring surgery is associated with significantly increased rates of operative complications as well as adverse nonsurgical events. Although the prevalence of COVID-19 has declined, these findings underscore the continued relevance of recent infection as a perioperative risk factor and highlight the need for future studies to refine timing guidelines. Based on the findings of this study, a 30-day delay in elective body-contouring procedures following COVID-19 infection may serve as a practical approach to postoperative risk mitigation.

## Supplemental Material

This article contains supplemental material located online at https://doi.org/10.1093/asjof/ojaf141.

## Supplementary Material

ojaf141_Supplementary_Data

## References

[1] Herzog I, Park J, Didzbalis CJ, Weisberger J, Tran BN, Lee ES. Assessing the impact of COVID-19 on facial aesthetic surgery search interest. Ann Plast Surg. 2023;90:S630–S633. doi: 10.1097/SAP.0000000000003499 36811485

[2] Lorenz FJ, Rothka AJ, Schopper HK, Lighthall JG. Impact of COVID-19 on facial plastic surgery volumes: a large database analysis of pre- and post-pandemic trends. Laryngoscope Investig Otolaryngol. 2024;9:e1292. doi: 10.1002/lio2.1292 PMC11166095 38864000

[3] Lem M, Kim JK, Pham JT, Tang CJ. Effect of the COVID-19 pandemic on global interest in plastic surgery. JPRAS Open. 2023;37:63–71. doi: 10.1016/j.jpra.2023.05.002 37360055 PMC10200276

[4] Aktas EH, Balci UD, Karacaoglu E. COVID pandemic aftermath: changing dynamics on cosmetic and aesthetic surgery demands. Aesthetic Plast Surg. 2023;47:1658–1665. doi: 10.1007/s00266-022-03231-9 36715726 PMC9886203

[5] Schlosser M, Signorelli H, Gregg W, Korwek K, Sands K. COVID-19 testing processes and patient protections for resumption of elective surgery. Am J Surg. 2021;221:49–52. doi: 10.1016/j.amjsurg.2020.07.009 32736801 PMC7367801

[6] Drygalski K, Płonowska E, Hady ZR, Głuszyńska P, Hady HR. Post-bariatric surgery abdominoplasty ameliorates psychological well-being in formerly obese patients: a cross-sectional recall study. J Clin Med. 2025;14:4025. doi: 10.3390/jcm14124025 40565771 PMC12194232

[7] Toma T, Harling L, Athanasiou T, Darzi A, Ashrafian H. Does body contouring after bariatric weight loss enhance quality of life? A systematic review of QOL studies. Obes Surg. 2018;28:3333. doi: 10.1007/s11695-018-3323-8 30069862 PMC6153583

[8] American Society of Plastic Surgeons. *2020 Plastic Surgery Statistics Report: ASPS National Clearinghouse of Plastic Surgery Procedural Statistics.* American Society of Plastic Surgeons; 2021. Accessed July 16, 2025. https://www.plasticsurgery.org/documents/news/statistics/2020/plastic-surgery-statistics-full-report-2020.pdf

[9] American Society of Plastic Surgeons. 2023 Plastic Surgery Statistics Report: ASPS National Clearinghouse of Plastic Surgery Procedural Statistics. American Society of Plastic Surgeons; 2024. Accessed July 16, 2025. https://www.plasticsurgery.org/documents/news/statistics/2023/plastic-surgery-statistics-report-2023.pdf

[10] American Society of Plastic Surgeons. *2024 Plastic Surgery Statistics Report: ASPS National Clearinghouse of Plastic Surgery Procedural Statistics.* American Society of Plastic Surgeons; 2025. Accessed July 16, 2025. https://www.plasticsurgery.org/documents/news/statistics/2024/plastic-surgery-statistics-report-2024.pdf

[11] Duggan RP, Lakatta A, Brondeel K, Donato DP, Phillips LG. Breast and body contouring outcomes after SARS-CoV-2 hospitalization. Plast Reconstr Surg Glob Open. 2022;10:24–25. doi: 10.1097/01.GOX.0000898432.07571.0b

[12] Wang F, Rothchild E, Ricci JA. The impact of prior infection with SARS-cov-2 on surgical outcomes in patients undergoing abdominal body contouring procedures. Ann Plast Surg. 2023;90:197–203. doi: 10.1097/SAP.0000000000003431 36752410

[13] von Elm E, Altman DG, Egger M, et al The Strengthening the Reporting of Observational Studies in Epidemiology (STROBE) statement: guidelines for reporting observational studies. J Clin Epidemiol. 2008;61:344–349. doi: 10.1016/j.jclinepi.2007.11.008 18313558

[14] Pitak-Arnnop P, Tangmanee C, Muangchan C, Meningaud JP, Neff A. Asymptomatic or mildly symptomatic COVID-19 patients with craniomaxillofacial injuries have an increased risk of surgical site infection. Br J Oral Maxillofac Surg. 2022;60:1118–1124. doi: 10.1016/j.bjoms.2022.05.009 35927146 PMC9155182

[15] Keske Ş, Altunok ES, Azak E, et al Impact of the COVID-19 pandemic on surgical site infections: a multi-center study evaluating incidence, pathogen distribution, and antimicrobial resistance patterns. Antimicrob Resist Infect Control. 2025;14:77. doi: 10.1186/s13756-025-01542-5 40598596 PMC12217381

[16] Davitt E, Davitt C, Mazer MB, Areti SS, Hotchkiss RS, Remy KE. COVID-19 disease and immune dysregulation. Best Pract Res Clin Haematol. 2022;35:101401. doi: 10.1016/j.beha.2022.101401 36494149 PMC9568269

[17] Teymourian H, ArianNik M, Mohit B, Massoudi N. A retrospective cohort study of the impact of COVID-19 infection control measures on surgical site infections in an academic hospital setting. Int Wound J. 2024;21:e14583. doi: 10.1111/iwj.14583 38453147 PMC10920026

[18] Schultheiß C, Willscher E, Paschold L, et al The IL-1β, IL-6, and TNF cytokine triad is associated with post-acute sequelae of COVID-19. Cell Rep Med. 2022;3:100663. doi: 10.1016/j.xcrm.2022.100663 35732153 PMC9214726

[19] Nie J, Zhou L, Tian W, et al Deep insight into cytokine storm: from pathogenesis to treatment. Signal Transduct Target Ther. 2025;10:112. doi: 10.1038/s41392-025-02178-y 40234407 PMC12000524

[20] Faraj SS, Jalal PJ. IL1β, IL-6, and TNF-α cytokines cooperate to modulate a complicated medical condition among COVID-19 patients: case-control study. Ann Med Surg. 2023;85:2291–2297. doi: 10.1097/MS9.0000000000000679 PMC10289607 37363608

[21] Nitsure M, Sarangi B, Shankar GH, et al Mechanisms of hypoxia in COVID-19 patients: a pathophysiologic reflection. Indian J Crit Care Med. 2020;24:967–970. doi: 10.5005/jp-journals-10071-23547 33281323 PMC7689135

[22] Salomão R, Assis V, de Sousa Neto IV, et al Involvement of matrix metalloproteinases in COVID-19: molecular targets, mechanisms, and insights for therapeutic interventions. Biology (Basel). 2023;12:843. doi: 10.3390/biology12060843 37372128 PMC10295079

[23] Mathew-Steiner SS, Roy S, Sen CK. Collagen in wound healing. Bioengineering (Basel). 2021;8:63. doi: 10.3390/bioengineering8050063 34064689 PMC8151502

[24] Biernacka A, Dobaczewski M, Frangogiannis NG. TGF-β signaling in fibrosis. Growth Factors. 2011;29:196–202. doi: 10.3109/08977194.2011.595714 21740331 PMC4408550

[25] Abbasifard M, Fakhrabadi AH, Bahremand F, Khorramdelazad H. Evaluation of the interaction between tumor growth factor-β and interferon type I pathways in patients with COVID-19: focusing on ages 1 to 90 years. BMC Infect Dis. 2023;23:248. doi: 10.1186/s12879-023-08225-9 37072722 PMC10112317

[26] Prasad M, Leon M, Lerman LO, Lerman A. Viral endothelial dysfunction: a unifying mechanism for COVID-19. Mayo Clin Proc. 2021;96:3099–3108. doi: 10.1016/j.mayocp.2021.06.027 34863398 PMC8373818

[27] Canale MP, Menghini R, Martelli E, Federici M. COVID-19–associated endothelial dysfunction and microvascular injury: from pathophysiology to clinical manifestations. Card Electrophysiol Clin. 2022;14:21–28. doi: 10.1016/j.ccep.2021.10.003 35221082 PMC8556628

[28] Vanderheiden A, Klein RS. Neuroinflammation and COVID-19. Curr Opin Neurobiol. 2022;76:102608. doi: 10.1016/j.conb.2022.102608 35863101 PMC9239981

[29] Freitas NL, Deus JVC, Sampaio K, et al Central nervous system and systemic inflammatory networks associated with acute neurological outcomes in COVID-19. Sci Rep. 2025;15:24154. doi: 10.1038/s41598-025-08632-9 40619452 PMC12230134

[30] Pinho-Ribeiro FA, Verri WA Jr, Chiu IM. Nociceptor sensory neuron-immune interactions in pain and inflammation. Trends Immunol. 2017;38:5–19. doi: 10.1016/j.it.2016.10.001 27793571 PMC5205568

[31] Shiers S, Ray PR, Wangzhou A, et al ACE2 and SCARF expression in human dorsal root ganglion nociceptors: implications for SARS-CoV-2 virus neurological effects. Pain. 2020;161:2494–2501. doi: 10.1097/j.pain.0000000000002051 32826754 PMC7572821

[32] Komaroff AL, Lipkin WI. ME/CFS and long COVID share similar symptoms and biological abnormalities: road map to the literature. Front Med (Lausanne). 2023;10:1187163. doi: 10.3389/fmed.2023.1187163 37342500 PMC10278546

[33] Gheorghita R, Soldanescu I, Lobiuc A, et al The knowns and unknowns of long COVID-19: from mechanisms to therapeutical approaches. Front Immunol. 2024;15:1344086. doi: 10.3389/fimmu.2024.1344086 38500880 PMC10944866

[34] McFadyen JD, Stevens H, Peter K. The emerging threat of (micro)thrombosis in COVID-19 and its therapeutic implications. Circ Res. 2020;127:571–587. doi: 10.1161/CIRCRESAHA.120.317447 32586214 PMC7386875

[35] Abou-Ismail MY, Diamond A, Kapoor S, Arafah Y, Nayak L. The hypercoagulable state in COVID-19: incidence, pathophysiology, and management. Thromb Res. 2020;194:101–115. doi: 10.1016/j.thromres.2020.06.029 32788101 PMC7305763

[36] Kichloo A, Dettloff K, Aljadah M, et al COVID-19 and hypercoagulability: a review. Clin Appl Thromb. 2020;26:1076029620962853. doi: 10.1177/1076029620962853 PMC7592310 33074732

[37] Griffin M, Akhavani MA, Muirhead N, Fleming AN, Soldin M. Risk of thromboembolism following body-contouring surgery after massive weight loss. Eplasty. 2015;15:e17. PMID: 26171089. 26171089 PMC4447099

[38] Colwell AS, Reish RG, Kuter DJ, Damjanovic B, Austen WG Jr, Fogerty AE. Abdominal contouring procedures increase activity of the coagulation cascade. Ann Plast Surg. 2012;69:129–133. doi: 10.1097/SAP.0b013e318226b38b 21734537

[39] Lip GYH, Stevens SM. *Venous Thromboembolism: Anticoagulation After Initial Management.* In: Mandel J, Douketis JD, Li H, Finlay G, eds. UpToDate; 2025. Accessed July 16, 2025. https://www.uptodate.com/contents/venous-thromboembolism-anticoagulation-after-initial-managementH28215493

[40] Bunch CM, Moore EE, Moore HB, et al Immuno-thrombotic complications of COVID-19: implications for timing of surgery and anticoagulation. Front Surg. 2022;9:889999. doi: 10.3389/fsurg.2022.889999 35599794 PMC9119324

[41] Singh TK, Zidar DA, McCrae K, et al A post-pandemic enigma: the cardiovascular impact of post-acute sequelae of SARS-CoV-2. Circ Res. 2023;132:1358–1373. doi: 10.1161/CIRCRESAHA.122.322228 37167358 PMC10171306

[42] Dani M, Dirksen A, Taraborrelli P, et al Autonomic dysfunction in ‘long COVID’: rationale, physiology and management strategies. Clin Med. 2021;21:e63–e67. doi: 10.7861/clinmed.2020-0896 PMC7850225 33243837

[43] Carlson FR Jr, Bosukonda D, Keck PC, Carlson WD. Multiorgan damage in patients with COVID-19: is the TGF- β/BMP pathway the missing link? JACC Basic Transl Sci. 2020;5:1145–1148. doi: 10.1016/j.jacbts.2020.09.003 32984657 PMC7508496

[44] Qudaisat IY, Toubasi AA, Obaid YY, Albustanji FH, Al-Harasis SM, AlOweidi AS. The delaying of elective surgeries after COVID-19 infection decreases postoperative complications. Asian J Surg. 2023;17:4308–4316. doi: 10.1016/j.asjsur.2023.05.002 PMC10188917 37225569

[45] Bryant JM, Boncyk CS, Rengel KF, et al Association of time to surgery after COVID-19 infection with risk of postoperative cardiovascular morbidity. JAMA Netw Open. 2022;5:e2246922. doi: 10.1001/jamanetworkopen.2022.46922 36515945 PMC9856239

[46] Codner JA, Archer RH, Lynde GC, Sharma J. Timing is everything: surgical outcomes for SARS-CoV-2 positive patients. World J Surg. 2023;47:437–444. doi: 10.1007/s00268-022-06814-4 36316514 PMC9628392

