# Supplementary material for: Recent COVID-19 Infection Increases Complication Risk After Body-Contouring Surgery
Source: Aesthet Surg J Open Forum. 2025 Oct 31;7:ojaf141. doi: 10.1093/asjof/ojaf141 (PMC12649762; doi:10.1093/asjof/ojaf141)
Supplement: ojaf141_Supplementary_Data [file ojaf141_supplementary_data.docx]

**Supplementary Table: ICD-10, CPT, and LOINC Codes**

| **Surgical Procedure** | **CPT Codes** |
| --- | --- |
| Mastopexy | 19316 |
| Panniculectomy | 15830 |
| Abdominoplasty | 15847 |
| Excision, excessive skin and subcutaneous tissue, of leg | 15833 |
| Excision, excessive skin and subcutaneous tissue, of submental fat pad | 15838 |
| Excision, excessive skin and subcutaneous tissue, of forearm or hand | 15837 |
| Excision, excessive skin and subcutaneous tissue, of hip | 15834 |
| Excision, excessive skin and subcutaneous tissue, of buttock | 15835 |
| Excision, excessive skin and subcutaneous tissue, of thigh | 15832 |
| Excision, excessive skin and subcutaneous tissue, of arm | 15836 |
| Mastectomy for gynecomastia | 19300 |
| **Outcomes, Complications, & COVID-19** | **ICD-10, CPT, & LOINC Codes** |
| Surgical Site Infection | T81.4, T81.4XXA |
| Wound Disruption | T81.3 |
| Acute Postoperative Pain | G89.18 |
| Hematoma | M96.84, M96.83, L76.3, L76.32 |
| Any Surgical Site Complications | T80-T88 |
| Seroma | L76.34 |
| Emergency Department Utilization | 1013711 |
| Anemia | D64 |
| COVID-19 Diagnosis | U07.1, 94500-2, 94500-6 |
